# Potential Drug Interaction Between Liraglutide and Clonazepam: A Case Report

**DOI:** 10.1155/crps/9100558

**Published:** 2025-02-11

**Authors:** Ali Kheradmand, Amir Mehrvar, Mohammad Abbasinazari

**Affiliations:** ^1^Department of Psychiatry, Taleghani Hospital Clinical Research Development Unit, Medical School, Shahid Beheshti University of Medical Sciences, Tehran, Iran; ^2^Musculoskeletal Injuries Research Center, Shahid Beheshti University of Medical Sciences, Tehran, Iran; ^3^Department of Clinical Pharmacy, School of Pharmacy, Shahid Beheshti University of Medical Sciences, Tehran, Iran

**Keywords:** aviophobia, clonazepam, drug interaction, liraglutide, panic attack

## Abstract

Aviophobia, that is, fear of flying, is a common type of anxiety disorder. Benzodiazepines are used in the treatment of anxiety. Liraglutide is a treatment for obesity or overweight in combination with weight-related comorbidity. We present the case of a 23-year-old woman with overweight and aviophobia, who used clonazepam as antianxious protection when flying. When taking liraglutide shortly before clonazepam the efficacy of clonazepam seemed absent as the patient experienced a panic attack at the airport. This finding suggests that liraglutide may interfere with clonazepam and reduce its effect. Further research is needed to establish this association.

## 1. Introduction

Aviophobia, the fear of flying, is a common situational-specific phobia classified under anxiety disorders in the Diagnostic and Statistical Manual of Mental Disorders, fifth edition (DSM-5) [1]. Individuals with aviophobia experience intense and irrational fear when traveling by airplane. The current treatment for aviophobia involves virtual reality exposure therapy, which may include diaphragmatic breathing exercises [2]. Limited literature exists on specific medications for treating aviophobia; however, benzodiazepines, beta-blockers, and selective serotonin reuptake inhibitors (SSRIs) have been mentioned [3]. Benzodiazepines, like clonazepam, work on gamma amino butyric acid (GABA) type A receptors in the cortex and limbic system to reduce anxiety and fear [4]. Like all medications, there are potential drug interactions between clonazepam and other drugs. A drug interaction occurs when the effects of one medication are altered by the prior or simultaneous administration of another medication [5]. Currently, there are no significant reports on drug interactions between clonazepam and liraglutide in the literature. Here we present a case that supports the idea that liraglutide may delay the oral absorption of clonazepam, reducing the antianxious effects of clonazepam.

## 2. Case Presentation

A 23-year-old Iranian woman presented at an outpatient clinic near Mehrabad Airport in Tehran, Iran, with symptoms of tachycardia, sweating, palpitations, feeling faint, lightheadedness, and rapid breathing. She was single and attended a medical university in Tehran. The patient had been diagnosed with panic disorder since the age of 20 years and had been prescribed sertraline 50 mg/day and clonazepam 1 mg/day in the past. Additionally, she had a fear of flying that started at the age of 21 years. She did not have any other medical conditions, but due to her family living far away, she had to travel by plane several times a year. Her psychiatrist advised her to take clonazepam 1 mg 2 h before flying to prevent panic attacks related to her fear of flying. She had followed this regimen for the past 2 years without experiencing any panic attacks during flights. The patient struggled with excess weight and 2 weeks earlier she consulted an endocrinologist for overweight treatment. With a body mass index (BMI) of 29 (normal value: 18.5–25, overweight: 25–30, and obese: >30) kg/m^2^, although without any weight-related comorbidity, the endocrinologist prescribed liraglutide 0.6 mg/day subcutaneously for the first week. The patient was advised to contact the doctor after a week to adjust the dosage if tolerated. She began liraglutide injections in the morning before breakfast. After a week, she reported good tolerance and increased satiety, expressing a desire to continue liraglutide. The physician recommended increasing the dose to 1.2 mg/day for the second week. Planning to fly back home, the patient took liraglutide at 8 A.M. before breakfast and clonazepam at 10 A.M. to manage potential panic attacks due to aviophobia. In the airport, at 11 A.M. she experienced tachycardia, palpitations, dizziness, and rapid breathing and was assisted by airport staff who called for medical help. Emergency technicians arrived and conducted an electrocardiography (EKG) and blood tests including high sensitivity troponin T, glucose, and potassium, all showing normal results. At 11:30 A.M. intravenous administration of 2 mg diazepam successfully sedated the patient, alleviating the symptoms. Despite no changes in clonazepam dosage or brand, she was puzzled by its lack of effectiveness in managing panic attacks as usual. The patient was discharged from the clinic on the same day without any complications. She did not experience any panic attacks on subsequent trips when she took clonazepam and refrained from using liraglutide in the mornings of her flights.

## 3. Discussion

There are pharmacologic options available to treat obesity that can help reduce appetite and/or decrease caloric intake. Liraglutide, a glucagon-like peptide-1 (GLP-1) receptor agonist, has been approved at a high dose as a pharmacologic treatment for obese children and adults as well as overweight individuals with at least one weight-related comorbidity [6]. The patient described here did not have any such comorbidity and liraglutide was, thus, prescribed off-label. The primary mechanism of action for liraglutide in treating obesity involves slowing gastric emptying, enhancing postprandial satiety, and reducing appetite and food intake by affecting the central nervous system [7]. In a review article, May and Schindler [8] have identified potential drug interactions of antidiabetic medications. They have determined that among antidiabetic agents, two categories, including incretin mimetics, for example, GLP-1 agonists, and sodium-glucose cotransporter-2 inhibitors, have a very low likelihood of interaction and are, therefore, recommended as an ideal combination from a clinical pharmacologic perspective. They have noted that so far, no clinically significant drug–drug interactions have been reported for liraglutide [8]. In a recent review, Calvarysky et al. [9] summarized data on drug–drug interactions between GLP-1 agonists and oral medications. The study concluded that only a few cases demonstrated a reduction in the maximum plasma concentration (*C*_max_) of the investigated oral medications. However, the study had several limitations: the number of healthy volunteers in each study was small and only a limited range of oral medications, such as warfarin, digoxin, and statins, were investigated. The review did not identify any case reports concerning drug interactions between GLP-1 agonists and benzodiazepines [9]. After the initial report of efficacy in panic disorder approximately 40 years ago, clonazepam, either alone or in combination with SSRIs, continues to be a crucial pharmacological option for managing panic disorder [4]. The oral absorption of clonazepam tablets is rapid, with a bioavailability exceeding 80%. *C*_max_ of clonazepam is typically achieved within 1–4 h. Due to its quick action, clonazepam is approved by the US Food and Drug Administration (FDA) for prevention of panic attacks [10]. Liraglutide has the effect of increasing peristalsis [11]. This action shortens the residence time of the drug in the intestine, which may result in incomplete absorption of clonazepam. In vitro assessments have shown that liraglutide exhibits a low potential for pharmacokinetic drug–drug interactions related to cytochrome P450 and plasma protein binding [12]. Thus, it seems that in the case presented here, the use of liraglutide affected the gastrointestinal absorption of clonazepam. While the clonazepam levels were not measured in this case, the immediate effect of clonazepam in handling an anxiety episode is crucial. Unfortunately, the desired outcome of clonazepam was not attained in the patient, leading to panic attack symptoms like tachycardia, palpitations, dizziness, lightheadedness, and rapid breathing. Perhaps if she had refrained from injecting liraglutide on the morning of the flight, she might not have experienced panic symptoms. It is noteworthy that the patient did not experience any panic attacks during subsequent flights when she did not administer liraglutide on the day of the flight. It is advisable to conduct human studies in the future to assess the coadministration of GLP-1 agonists such as liraglutide with medications necessitating rapid absorption.

## 4. Conclusion

Our report highlights the potential interaction between liraglutide and clonazepam. This discovery could be advantageous for individuals needing prompt action of clonazepam during panic-inducing situations. Nonetheless, further research is required to determine, explore, and optimize this potential interaction.

## Data Availability

The data that support the findings of this study are available from the corresponding author upon reasonable request.
